# P2Y_6_ Receptor-Mediated Proinflammatory Signaling in Human Bronchial Epithelia

**DOI:** 10.1371/journal.pone.0106235

**Published:** 2014-09-22

**Authors:** Yuan Hao, Jocelyn F. Liang, Alison W. Chow, Wing-tai Cheung, Wing-hung Ko

**Affiliations:** School of Biomedical Sciences, The Chinese University of Hong Kong, Shatin, NT, Hong Kong, China; University of Colorado, Denver, United States of America

## Abstract

P2Y receptors are expressed in virtually all epithelia and are responsible for the control of fluid and electrolyte transport. In asthmatic inflammation, the bronchial epithelia are damaged by eosinophil-derived, highly toxic cationic proteins, such as major basic protein (MBP). Consequently, extracellular nucleotides are released into the extracellular space from airway epithelial cells, and act in an autocrine or paracrine fashion to regulate immune functions. Our data show damage to the human bronchial epithelial cell line, 16HBE14o-, by poly-L-arginine-induced UDP release into the extracellular medium. Activation of P2Y_6_ receptor by its natural ligand, UDP, or its specific agonist, MRS 2693, led to the production of two proinflammatory cytokines, interleukin (IL)-6 and IL-8. This may have resulted from increased IL-6 and IL-8 mRNA expression, and activation of p38 and ERK1/2 MAPK, and NF-*κ*B pathways. Our previous study demonstrated that UDP stimulated transepithelial Cl^−^ secretion via both Ca^2+^- and cAMP-dependent pathways in 16HBE14o- epithelia. This was further confirmed in this study by simultaneous imaging of Ca^2+^ and cAMP levels in single cells using the Fura-2 fluorescence technique and a FRET-based approach, respectively. Moreover, the P2Y_6_ receptor-mediated production of IL-6 and IL-8 was found to be dependent on Ca^2+^, but not the cAMP/PKA pathway. Together, these studies show that nucleotides released during the airway inflammatory processes will activate P2Y_6_ receptors, which will lead to further release of inflammatory cytokines. The secretion of cytokines and the formation of such “cytokine networks” play an important role in sustaining the airway inflammatory disease.

## Introduction

Chloride (Cl^−^) secretion can be modulated in the airway through activation of multiple P2Y receptors. The bronchial epithelial cell line, 16HBE14o-, expresses different P2Y receptor subtypes, including P2Y_6_ [1]. Our laboratory recently demonstrated unequivocally that activation of the P2Y_6_ receptor stimulated Cl^−^ secretion via both Ca^2+^- and cAMP-dependent pathways through the activation of the calcium-activated Cl^−^ channel (CaCC) and cystic fibrosis transmembrane conductance regulator (CFTR) in human bronchial epithelia [1]. In addition to ion transport regulation in various epithelia, P2Y receptors have been implicated as important components in the initiation, amplification, and spread of acute inflammation, and in the down-regulation of chronic inflammation [2], [3]. It has been reported that extracellular nucleotides trigger the secretion of cationic protein from human eosinophils, possibly via P2Y_2_ receptors. More recent evidence suggests that P2Y_6_ receptor may also be a key component in promoting inflammation [4], [5]. For example, the release of IL-8 has been shown to be due to activation of the P2Y_6_, P2X_1_, and P2X_7_ receptors [6]. It was recently shown for the first time that P2Y_6_ receptors modulated the release of CCL20 chemokines and IL-8 from human nasal epithelial cells, thus possibly altering immune cell recruitment in the airway [7]. The specific agonist for the P2Y_6_ receptor, UDP, stimulated the production of proinflammatory cytokines in retinal pigment epithelial cells [8] and monocytic cells [9]. In an ulcerative colitis mouse model, mRNA and protein expression of both the P2Y_2_ and P2Y_6_ receptors were shown to be up-regulated in the inflammatory response of intestinal epithelial cells. Stimulation of these cells with the P2Y_6_ receptor agonist, UDP, was found to result in the increased expression and release of IL-8 [10]. Idzko *et al*. performed a series of elegant *in vivo* experiments, providing the first evidence showing that extracellular ATP is indeed an important mediator in the pathogenesis of asthma in a mouse model [11]. Moreover, mRNA for several P2X and P2Y receptors, including P2Y_6_, has been detected in the lungs of asthmatic mice.

During airway inflammation, damage to the surface epithelium is due to the secretion of eosinophil-derived, highly toxic cationic proteins, such as the major basic protein (MBP) [12]. Our previous study made use of a cellular model of asthmatic inflammation in airway epithelial cells by challenging bronchial epithelial cells, 16HBE14o-, with a highly charged cationic protein, poly-L-arginine, as a surrogate of MBP. We found that the epithelium itself was responsible for the synthesis and release of at least five proinflammatory cytokines, including IL-6 and IL-8, via the NF-*κ*B, p38 and ERK1/2 MAPK pathways [13]. This may cause the selective recruitment, retention, and accumulation of various inflammatory cells during inflammation.

A recent study demonstrated that UDP is released from cortical slices, and its concentration could be quantified by a newly developed fluorescent dye, Probe-1 [4]. Therefore, it is possible that during asthmatic inflammation, activation of P2Y_6_ receptors via the released nucleotides may play a proinflammatory role in stimulating the biosynthetic release of various cytokines. Because the functions and mechanisms of P2Y_6_ receptor in modulating the airway inflammatory response remain largely unknown, we examined the proinflammatory role of P2Y_6_ receptors in human bronchial epithelia. The results of the study showed that a significant amount of UDP was released upon cellular damage by poly-L-arginine, and the activation of P2Y_6_ receptors by UDP significantly induced IL-6 and IL-8 release in 16HBE14o- cells. The underlying cellular pathways mainly involved Ca^2+^, p38 and ERK1/2 MAPK, and NF-*κ*B.

## Materials and Methods

### Cells and Reagents

The immortalized cell line, 16HBE14o-, was derived from bronchial surface epithelial cells [14]. Standard culture techniques were used as described previously [1], [13]. In brief, cells were maintained in Minimum Essential Medium with Earle’s salts supplemented with 10% (v/v) fetal bovine serum, 1% (v/v) L-glutamine, 100 IU/ml penicillin, and 100 µg/ml streptomycin. Cells were cultured on plastic flasks coated with fibronectin and collagen (BD Biosciences, San Jose, CA, USA) and were incubated in humidified 95% air-5% CO_2_ at 37°C. Primary human bronchial epithelial (HBE) cells were obtained from ScienCell Research Laboratories (Carlsbad, CA, USA) and maintained in Bronchial Epithelial Cell Medium (ScienCell Research Laboratories). All other general laboratory reagents were obtained from Sigma-Aldrich (St. Louis, MO, USA), and all cell culture reagents were obtained from Invitrogen (Carlsbad, CA, USA). Before the use of UDP, this nucleotide (10 mM) was incubated (1 h at 37°C) in HEPES-buffered saline containing hexokinase (10 IU/ml; Boehringer, Mannheim, Germany) and 22 mM D-glucose in order to remove contaminating nucleotide triphosphates. The resulting solution was then aliquoted and stored at −20°C [15].

### Lactate Dehydrogenase (LDH) Release from 16HBE14o- Cells

The 16HBE14o- cells were incubated with various concentrations of poly-L-arginine for 3 h. Cell culture supernatants were collected and centrifuged at 3000×*g* for 10 min at 4°C. Damage of the 16HBE14o- cells by poly-L-arginine was assessed by release of the cytosolic enzyme LDH into the extracellular medium. The adherent cells were lysed with 100 µl 0.05% Triton X-100 in bovine serum albumin (BSA) for the determination of maximum LDH release. The LDH activity in cell culture supernatants and the cell lysate was assayed using the Cytotoxicity Detection Kit^Plus^ (LDH) (Roche, Basel, Switzerland), according to the manufacturer’s instructions.

### ELISA

Quantification of IL-6 and IL-8 secretion was done with an enzyme-linked immunosorbent assay (ELISA). Cells were grown in 24-well culture plates. Cell-free supernatants were collected from control and treated cells and analyzed using a commercially available ELISA kit specific for IL-6 (eBioscience, San Diego, CA, USA) and IL-8 (BD Biosciences, San Diego, CA, USA), according to the manufacturers’ protocol. All measurements were made in duplicate.

### Measurement of UDP Release from 16HBE14o- Cells

The concentration of UDP in the extracellular medium was measured using a method similar to that described by Kim *et al*. [4]. Briefly, cells were cultured in 24-well plates until confluent. The medium was replaced with HEPES-buffered solution [130 mM NaCl, 5 mM KCl, 1.5 mM CaCl_2_, 1 mM MgCl_2_, 10 mM glucose, and 10 mM HEPES (pH 7.4)] for 1 h, and then the cells were stimulated with poly-L-arginine for 1 h. The levels of UDP secreted into the solution were measured using Probe-1 provided by Dr. Juyoung Yoon (Department of Chemistry and Nano Science, Ewha Woman’s University, Suwon, Korea). This probe specifically detects UDP (with lower sensitivity for UTP), but not other nucleotides, such as ATP, ADP, and UMP/uridine [16]. Samples were mixed with Probe-1 (final concentration was 10 µM) and fluorescence levels were measured at 485/535 nm (excitation/emission) with a DTX 880 multimode detector (Beckman Coulter, Hong Kong, China).

### RNA Extraction and Quantitative Real-Time PCR (qRT-PCR)

Total RNA was extracted with TRIzol Reagent (Invitrogen) according to the manufacturer’s instructions as previously described. RNA was reverse transcribed to cDNA using an iScript^™^ Synthesis Kit (Bio-Rad Laboratories, Hercules, CA, USA). Real-time PCR was performed with an Applied Biosystems Power SYBR Green PCR Master Mix (Invitrogen) on an iCycler thermal cycler (Bio-Rad Laboratories). Primer sequences were as follows: IL-6 forward primer, 5′-GCACTGGCAGAAAACAACCT-3′, reverse primer, 5′-TCAAACTCCAAAAGACCAGTGA-3′; IL-8 forward primer, 5′-CCAACACAGAAATTATTGTAAAGC-3′, reverse primer, 5′-TGAATTCTCAGCCCTCTTCAA-3′; GAPDH forward primer, 5′-TGCACCACCAACTGCTTAGC-3′, reverse primer, 5′-GGCATGGACTGTGGTCATGAG-3′. Relative expression of IL-6 and IL-8 was normalized to GAPDH and determined using the Pfaffl method. A standard curve of threshold values (Ct) versus concentration was plotted to obtain the efficiency of each PCR reaction. This efficiency (E) can be obtained by the formula 10−(1/slope), with an acceptable range of 1.8–2.2. Each PCR run included a control with no template and a sample without reverse transcriptase. All measurements were performed in duplicate. PCR reactions for human P2Y1, P2Y2, P2Y4 and P2Y6 receptors used the primers described previously [1].

### Western Blot Analysis

Cells grown in a 6-well plate were lysed in CytoBuster Protein Extraction Reagent (Merck, Darmstadt, Germany) supplemented with protease inhibitor and phosphatase inhibitor cocktail (Calbiochem, Darmstadt, Germany). The cell lysate was collected and the supernatant harvested after centrifugation at 20,000×*g* for 20 min at 4°C. Total protein content in each sample was determined using the Bradford assay (Bio-Rad). Forty micrograms of protein were used for the western blot and separated by 12% SDS-PAGE. Separated proteins were electroblotted onto a polyvinylidene fluoride (PVDF) membrane (Immobilon-P; Millipore Corporation, Bedford, MA, USA) using a wet transfer system (Bio-Rad). Membranes were blocked for 30 min at room temperature using 1% BSA in PBS containing 0.05% Tween 20 and incubated overnight at 4°C with the following polyclonal antibodies: goat polyclonal anti-P2RY_1_ (Santa Cruz Biotechnology, Dallas, TX, USA, 1∶500), rabbit polyclonal anti-P2RY_2_ (Santa Cruz Biotechnology, 1∶300), anti-P2YR_4_ (Alomone Labs, Jerusalem, Israel, 1∶300) and rabbit monoclonal anti-P2YR_6_ (Epitomics, Burlingame, CA, USA, 1∶1000); anti-phospho-p38 MAPK (Cell Signaling Technology, Beverly, MA, USA, 1∶1000), anti-p38 MAPK (Cell Signaling Technology, 1∶1000), anti-p44/42 MAPK (ERK1/2; Cell Signaling Technology, 1∶1000), anti-phospho-p44/42 MAPK (ERK1/2), (Cell Signaling Technology, 1∶1000); anti-GAPDH (Ambion, Austin, TX, USA, 1∶500,000). The positions of positive bands were detected by incubation with horseradish peroxidase-conjugated anti-rabbit or anti-mouse IgG (Dako, Glostrup, Denmark) and visualized using an enhanced chemiluminescence detection system (Millipore). Their apparent molecular masses were calculated based on prestained SDS-PAGE mid-range protein markers (Hou-Bio Life Technologies, Hong Kong, China).

### Simultaneous Imaging of Ca^2+^ and cAMP Levels

Imaging experiments were conducted using an approach similar to that described by Landa *et al*. [17]. Real-time cAMP changes in living cells were monitored using CFP-Epac-YPF, an Epac-based polypeptide FRET reporter [18], as previously described [19]. FRET imaging experiments were performed using the MetaFluor Imaging System (with FRET module) (Molecular Devices, LLC, Sunnyvale, CA, USA). The 16HBE14o- cells were grown on Transwell-COL membranes (Costar, Cambridge, MA, USA) to form polarized monolayers [1], which were transfected with the Epac-based cAMP sensor. Transfected cells were loaded with Fura-2-AM (3 µM, 45 min) and then transferred to a closed perfusion chamber mounted on an inverted microscope (Olympus IX70, Center Valley, PA, USA) equipped with a 20× water immersion objective (numerical aperture 0.6). Cells were excited at 340 nm, 380 nm, and 436 nm. CFP and YFP images were simultaneously recorded by the imaging system equipped with the Photometrics DV^2^ emission splitting system (Photometrics, Tucson, AZ, USA) including two emission filters (470/30 nm for CFP and 535/30 nm for FRET), and a digitally cooled CCD camera (Quantix; Photometrics). Acquired fluorescence images were background subtracted, and real-time cAMP changes were represented by normalized CFP/FRET emission ratios similar to that described by Li *et al*. [20]. Fura-2 ratio images (>510 nm) were also recorded to represent changes in [Ca^2+^]_i_. For some experiments, only Fura-2 images were acquired in cells grown on glass coverslips.

### NF-*κ*B Translocation Assay

Cells were grown in black 96-well glass bottom plates (Costar). Measurement of NF-*κ*B translocation from the cytoplasm to the nucleus was performed as previously described [13] using a Cellomics NF-*κ*B Activation Kit^™^ (Thermo Fisher Scientific, Pittsburgh, PA, USA) according to the manufacturer’s protocol. The nucleus was immunostained with Hoechst 33342, and NF-*κ*B was detected with an NF-*κ*B primary antibody and Alexa Fluor 488-conjugated goat anti-rabbit IgG secondary antibody. Microscopic images were captured with the MetaFluor Imaging System as described above. Image analysis was performed using NIH ImageJ software (v.3.91, http://rsbweb.nih.gov/ij/) as described by Noursadeghi et al. [21]. NF-*κ*B nuclear translocation was determined as an increase in the nucleus∶cytoplasm ratio.

### Data Analysis

Pooled data were presented as means ± standard errors (S.E.), and values of *n* refer to the number of experiments in each group. Statistical comparisons between original data before normalization were performed using Student’s *t*-test and analysis of variance (ANOVA), where appropriate, with *p*<0.05 considered significant.

## Results

### Expression of P2Y Receptor Subtypes in 16HBE14o- Cells and Primary HBE Cells

**Figure 1:**
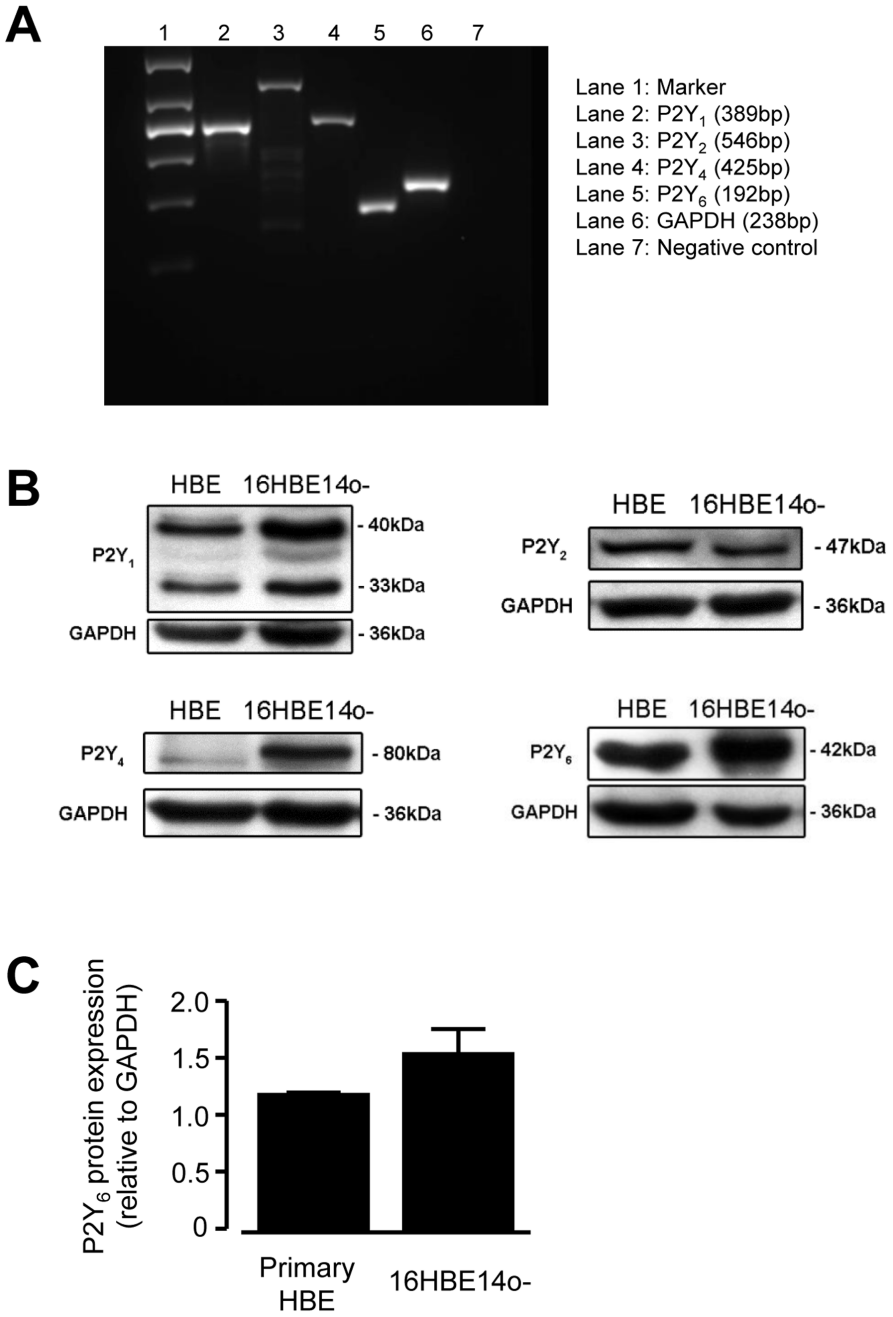
Detection of mRNA and protein expression of P2Y receptor subtypes. (A) Amplification products of the predicted size were found after RT-PCR of primary human bronchial epithelial (HBE) cells. Representative agarose gel showing the products of P2Y_1_ (lane 2, 389 bp), P2Y_2_ (lane 3, 546 bp), P2Y_4_ (lane 4, 425 bp), and P2Y_6_ (lane5, 192 bp) subtype-specific PCR products. Lane 1, 100 bp molecular weight standard; lane 6, negative control (without reverse transcription). (B) Western blots of P2Y receptor subtypes in primary HBE cells and the 16HBE14o- cell line. Expression of P2Y_1_ (40 kDa), P2Y_2_ (47 kDa), P2Y_4_ (80 kDa), and P2Y_6_ (42 kDa) receptors are shown (lane 1). For P2Y_1_, an additional lower molecular weight product (33 kDa) was also found. (C) P2Y_6_ receptor protein expression was not statistically different between primary HBE cells and the 16HBE14o- cell line (*n* = 3, *p*<0.05, Student’s *t*-test).

**Figure 2:**
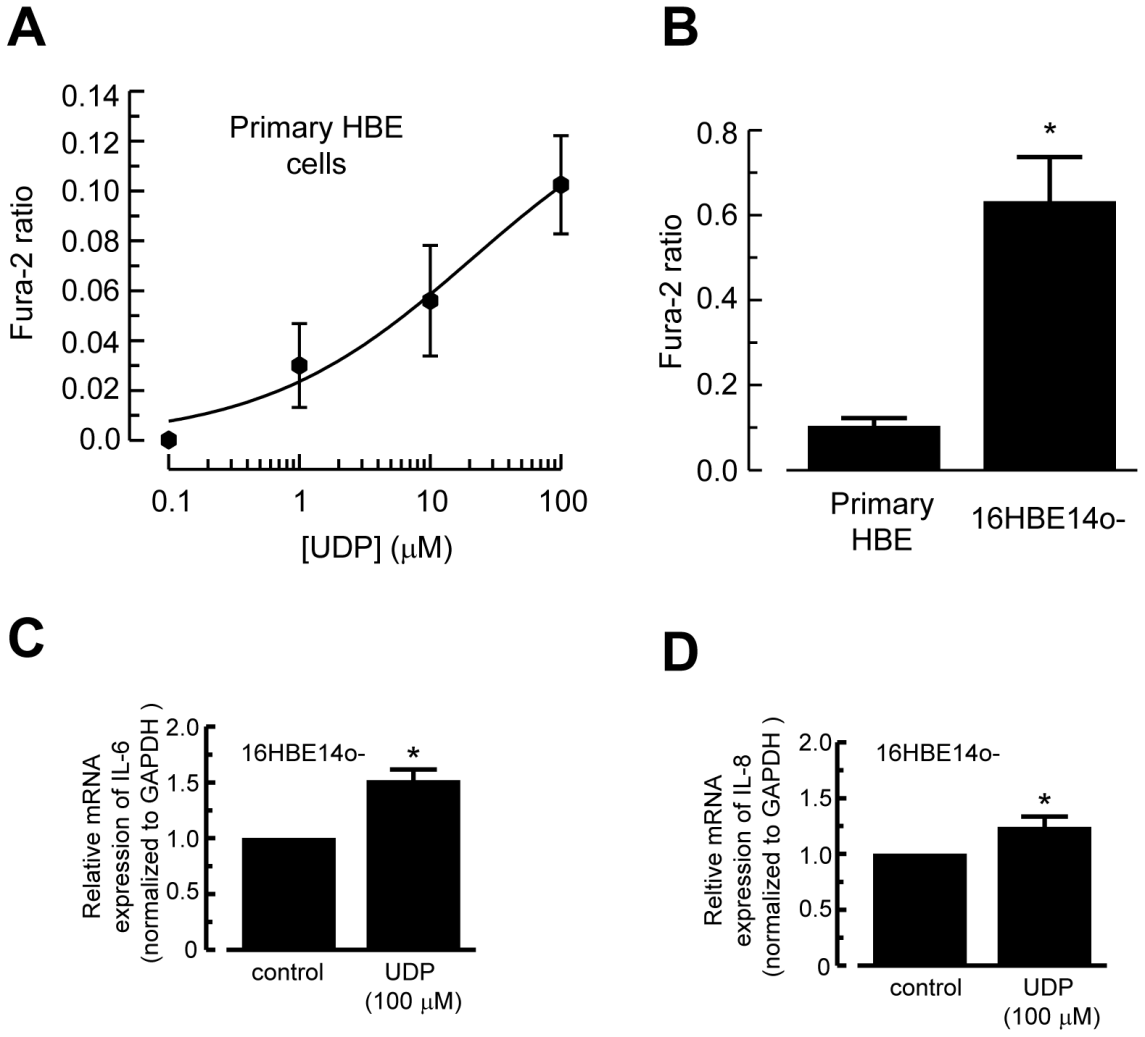
UDP increased [Ca^2+^]_i_, IL-6, and IL-8 mRNA expression. (A) Concentration-response of UDP upon changes in [Ca^2+^]_i_ in primary HBE cells. The cells were stimulated with different concentrations of UDP, and the Fura-2 ratio was quantified and plotted against the concentration of UDP (*n* = 3–7). Each data point represents the mean ± S.E. (B) The effect of 100 µM UDP on [Ca^2+^]_i_ in 16HBE14o- cells was significantly larger than that of primary HBE cells. (*p<0.05, Student’s *t*-test, *n* = 8). (C–D) The qRT-PCR analysis of mRNA for IL-6 (C) and IL-8 (D). Epithelia were treated with UDP (100 µM) for 6 h. Relative expression of IL-6 and IL-8 was normalized to GAPDH and is shown as fold change relative to untreated controls. Each column represents the mean ± S.E. (*n* = 5).

We have demonstrated previously that 16HBE14o- cells expressed P2Y_1_, P2Y_2_, P2Y_4_, and P2Y_6_ mRNA and protein [1]. In this study, qRT-PCR indicated that primary HBE cells expressed the mRNA for these four P2Y receptor subtypes (Fig. 1A). Protein expression of P2Y receptor subtypes in 16HBE14o- and primary HBE cells was examined using western blot analysis. In each case, a major protein band was found with an apparent molecular mass of 40 kDa for P2Y_1_, 47 kDa for P2Y_2_, 80 kDa for P2Y_4_, and 42 kDa for P2Y_6_ (Fig. 1B). Detection of these protein bands was specific as they were abolished by prior preabsorption of the antibodies with their respective control antigens for 2 h at 4°C (data not shown). Therefore, the same P2Y receptor subtypes were expressed in both cell types. In addition, expression of the P2Y_6_ receptor in primary HBE cells did not differ when compared with 16HBE14o- cells (Fig. 1C). When primary HBE cells were exposed to increasing concentrations (0.1–100 µM) of UDP, the natural ligand for P2Y_6_ receptor, concentration dependent increases in [Ca^2+^]_i_ were observed (Fig. 2A). However, the 100 µM UDP-activated calcium increase in primary HBE cells was significantly lower than that of 16HBE14o- cells (Fig. 2B). Together, the results indicated that both cell types expressed functional P2Y_6_ receptors with differential release of Ca^2+^ in the presence of UDP.

### UDP Increased IL-6 and IL-8 mRNA Expression

To determine whether UDP induced IL-6 and IL-8 mRNA expression, we quantitated IL-6, IL-8, and GAPDH mRNA expression in 16HBE14o- cells by RT-PCR. After stimulation of the cells with UDP (100 µM) for 6 h, there was an increase in IL-6 (Fig. 2C) and IL-8 (Fig. 2D) mRNA expression relative to GAPDH, indicating UDP increased synthesis of IL-6 and IL-8 mRNA.

### Involvement of P2Y_6_ Receptor in Poly-L-Arginine-Stimulated IL-6 and IL-8 Release

**Figure 3:**
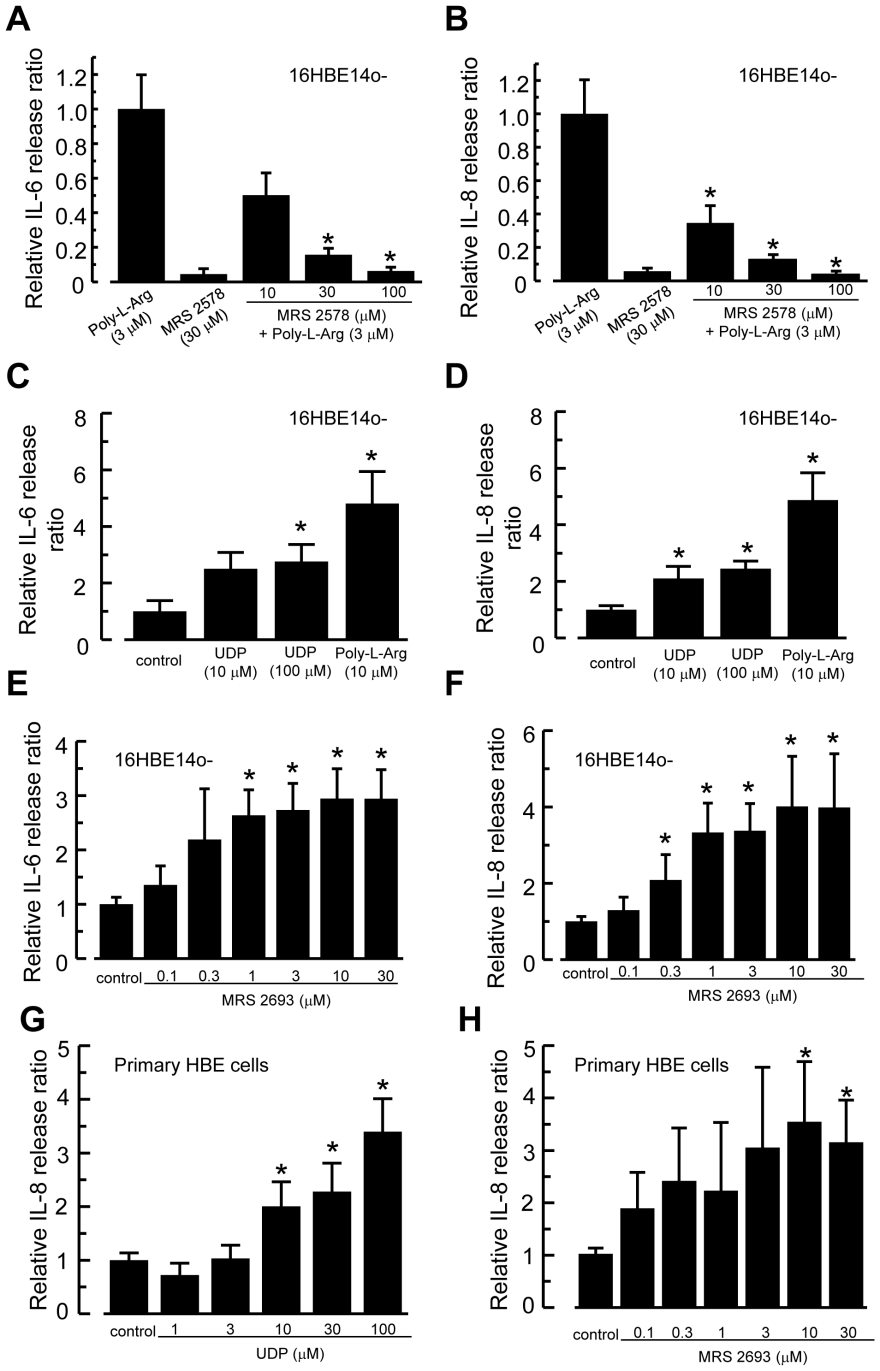
Involvement of P2Y_6_ receptors in the poly-L-arginine-induced cytokine response. Human bronchial epithelial cells (16HBE14o-) were treated with a P2Y_6_ receptor antagonist, MRS 2578, alone or with poly-L-arginine for 3 h in the absence or presence of different concentrations of the blocker. Cell-free supernatants were assayed by ELISA for IL-6 (A) and IL-8 (B). Levels of IL-6 and IL-8 were corrected with vehicle control alone and normalized to 3 µM poly-L-arginine. Each column represents the mean ± S.E. (*n* = 5–7). Statistically significant inhibitory effects compared with poly-L-arginine-treated controls are marked with an asterisk (*p<0.05, Student’s *t*-test). (C-F) Similar experiments were performed by stimulating 16HBE14o- cells with the natural ligand for the P2Y_6_ receptor, UDP (10 µM and 100 µM), or a specific P2Y_6_ agonist, MRS 2693. Cell supernatants were analyzed for IL-6 (C, E) and IL-8 (D, F). Each column represents the mean ± S.E. (*n* = 4–8). Statistically significant stimulatory effects compared with vehicle control (normalized to 1) are marked with an asterisk (*p<0.05, Student’s *t*-test). (G, H) UDP or MRS 2693 also stimulated IL-8 release in primary HBE cells (*n* = 5–7).

Our previous study demonstrated that 16HBE14o- epithelia can be “chemically injured” by exposing to the cationic polypeptide, poly-L-arginine, leading to secretion of both IL-6 and IL-8 into the extracellular medium. We further demonstrated cellular damage in this study by measuring release of cytosolic enzyme LDH from 16HBE14o- cells treated with poly-L-arginine. Exposure of cultured epithelia to 1, 3, 10, and 30 µM poly-L-arginine for 3 h stimulated a concentration dependent increase in LDH release from 0.04±0.03 U/ml (control) to 0.14±0.05, 0.72±0.32, 0.91±0.31, and 1.55±0.35 U/ml, respectively (*n* = 3–7). The maximum LDH release from the cell lysate was 7.87±1.06 U/ml. There was a significant LDH release induced by poly-L-arginine starting from 3 µM, with a viability of 87.74±3.71% (*n* = 4). However, there was no significant difference in cell viability from 3 to 30 µM (*p*>0.05, ANOVA, with Dunnett T3 correction). Therefore, we hypothesized that nucleotides were released during cellular damage and then act on membrane P2Y_6_ receptors expressed on 16HBE14o- cells, causing release of IL-6 and IL-8. To demonstrate the involvement of P2Y_6_ receptors, the inhibitory effects of a specific P2Y_6_ antagonist, MRS 2578, on poly-L-arginine-induced IL-6 and IL-8 secretion were investigated. Cells were stimulated with 3 µM poly-L-arginine for 3 h in the absence or presence of MRS2578, and the release of IL-6 and IL-8 was quantified by ELISA. MRS 2578 blocked poly-L-arginine-induced IL-6 (Fig. 3A) and IL-8 (Fig. 3B) secretion in a concentration dependent manner. MRS 2578 inhibited IL-6 and IL-8 release induced by poly-L-arginine most significantly at 100 µM, where it reduced the IL-6 and IL-8 release ratios to 0.06±0.02 and 0.04±0.02, respectively, when compared to poly-L-arginine-treated controls. MRS 2578 alone did not have any significant effect on basal IL-6 and IL-8 levels. Together, these results suggest the involvement of P2Y_6_ receptors in mediating release of IL-6 and IL-8.

To demonstrate that activation of P2Y_6_ receptors leads to release of IL-6 and IL-8 in normal cells, the cells were stimulated with a natural ligand of P2Y_6_ receptors, UDP, for 6 h. Fig. 3C and 3D show that hexokinase-treated UDP induced IL-6 and IL-8 release in a concentration dependent manner. Poly-L-arginine treatment (10 µM, 3 h) was used as a positive control for comparison. Hexokinase alone did not have an effect on IL-6 and IL-8 release (data not shown). At 100 µM, UDP increased IL-6 and IL-8 levels by 2.8±0.6 and 2.5±0.3 fold, respectively. As shown in Fig. 3E and 3F, similar stimulatory effects on IL-6 and IL-8 release were observed when cells were treated with a highly selective P2Y_6_ agonist, MRS 2693 for 6 h. These results suggest that release of IL-6 and IL-8 caused by poly-L-arginine-induced cellular damage involves P2Y_6_ receptor activation in human bronchial epithelia. Experiments were also performed in primary human bronchial epithelial (HBE) cells. Stimulating the cells with different concentrations of UDP or MRS 2693 for 6 h resulted in a significant increase in IL-8 release (Fig. 3G, 3H). There was no detectable increase in IL-6 release when the primary cells were treated with 100 µM UDP or 30 µM MRS 2693 as compared with vehicle control (*n* = 5–7). However, when the primary HBE cells were treated with 1 µM poly-L-arginine, there was a 2.53±0.84 and 5.68±1.98 fold increase in IL-6 and IL-8 release, respectively, when compared with control (*n* = 5, *p*<0.05). In a separate experiment, 16HBE14o- cells were treated with poly-L-arginine (10 µM) alone or together with UDP (100 µM) for 6 h. Poly-L-arginine induced a marked increase in IL-6 (control, 1.0±0.3 fold; treated, 11.4±3.5 fold, *n* = 5) and IL-8 secretion (control, 1.0±0.2 fold; treated, 10.9±3.0 fold, *n* = 4). However, the presence of UDP did not further increase IL-6 levels (5.7±2.3 fold; ANOVA, *p*>0.05) and IL-8 secretion (4.3±1.9 fold; ANOVA, *p*>0.05) when compared to that of poly-L-arginine-treated cells.

### UDP Released from Cells Damaged by Poly-L-Arginine

**Figure 4:**
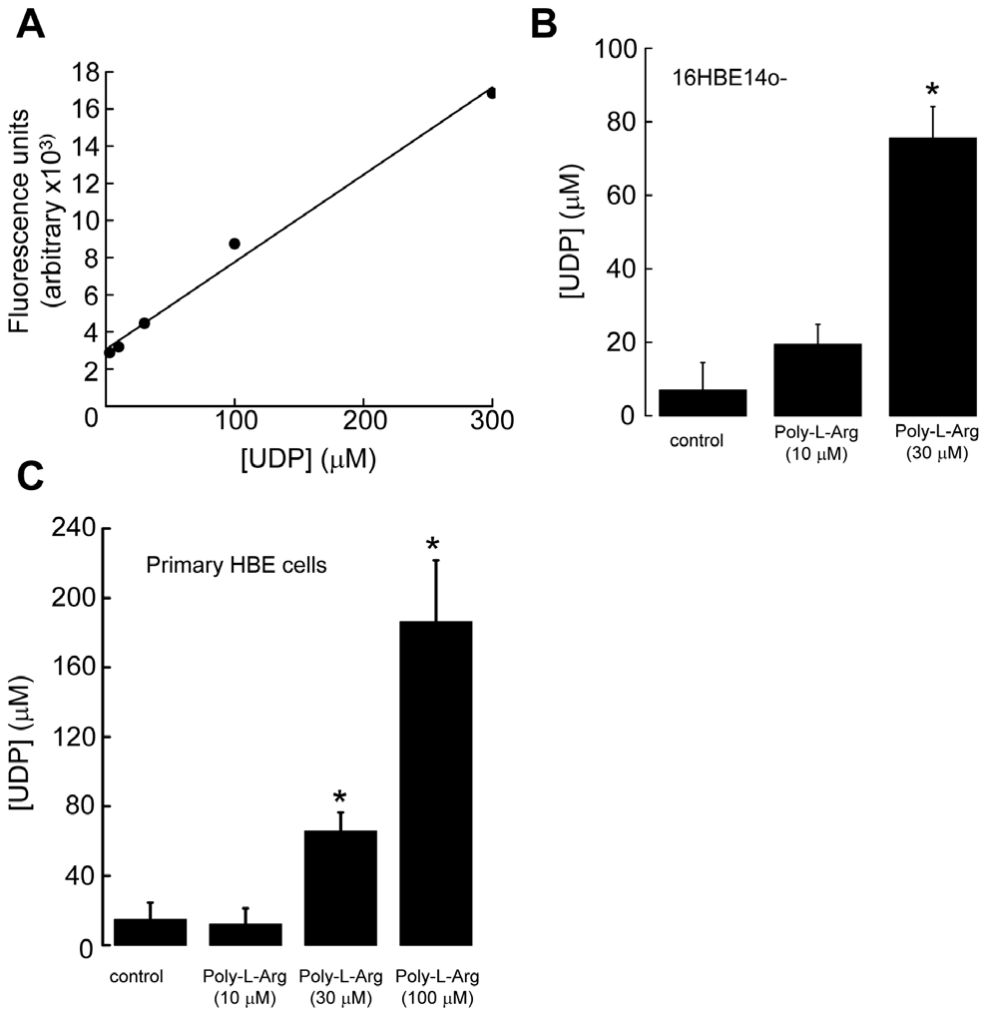
Measurement of UDP release from 16HBE14o- cells and primary HBE cells damaged by poly-L-arginine. (A) Probe-1 fluorescence emission intensity as a function of UDP concentration. The line represents a linear regression applied to the data, with a *R^2^* of 0.9906. (B) 16HBE14o- cells were incubated in HEPES-buffered saline in the presence of poly-L-arginine (10 µM and 30 µM) for 1 h. Levels of secreted UDP were measured using Probe-1 as described. Each column represents the mean ± S.E. (*n* = 4). Statistically significant effects compared with vehicle control are marked with an asterisk (*p<0.05, Student’s *t*-test). (C) Similar experiments were performed in primary HBE cells (*n* = 4).

Because the P2Y_6_ receptor is activated by extracellular UDP, and uridine nucleotides are known to be released by human astrocytoma cells induced by mechanical stress, we examined whether UDP is released after damage of bronchial epithelia by poly-L-arginine. Recently, a new and convenient method has been developed to detect the cellular release of UDP in cortical slices, which made use of a fluorescent probe known as Probe-1. This probe specifically detects UDP and UTP (with a low sensitivity), but not other nucleotides such as ATP, ADP, and UMP/uridine. Fig. 4A shows a typical calibration curve of Probe-1, demonstrating good linearity between the emitted fluorescence and the concentration of UDP in buffer, with a high correlation coefficient (*R^2^* = 0.9906). After exposing 16HBE14o- cells with 10 µM poly-L-arginine for 1 h, ∼19.4±5.5 µM UDP was detected (2.8±0.8 fold increase) as shown in Fig. 4B. The extracellular concentration of UDP further increased to ∼75.5±8.6 µM (11.0±1.3 fold increase) when the epithelia were exposed to a higher concentration of poly-L-arginine (30 µM). UDP release data of similar magnitude were obtained when primary HBE cells were damaged by poly-L-arginine for 1 h (Fig. 4C). Together, these data suggest that exposing epithelia to poly-L-arginine resulted in a significant amount of UDP being released into the extracellular medium in both a cell line and primary cultured cells.

### Involvement of p38 MAPK in P2Y_6_ Receptor-Mediated IL-6 and IL-8 Release

**Figure 5:**
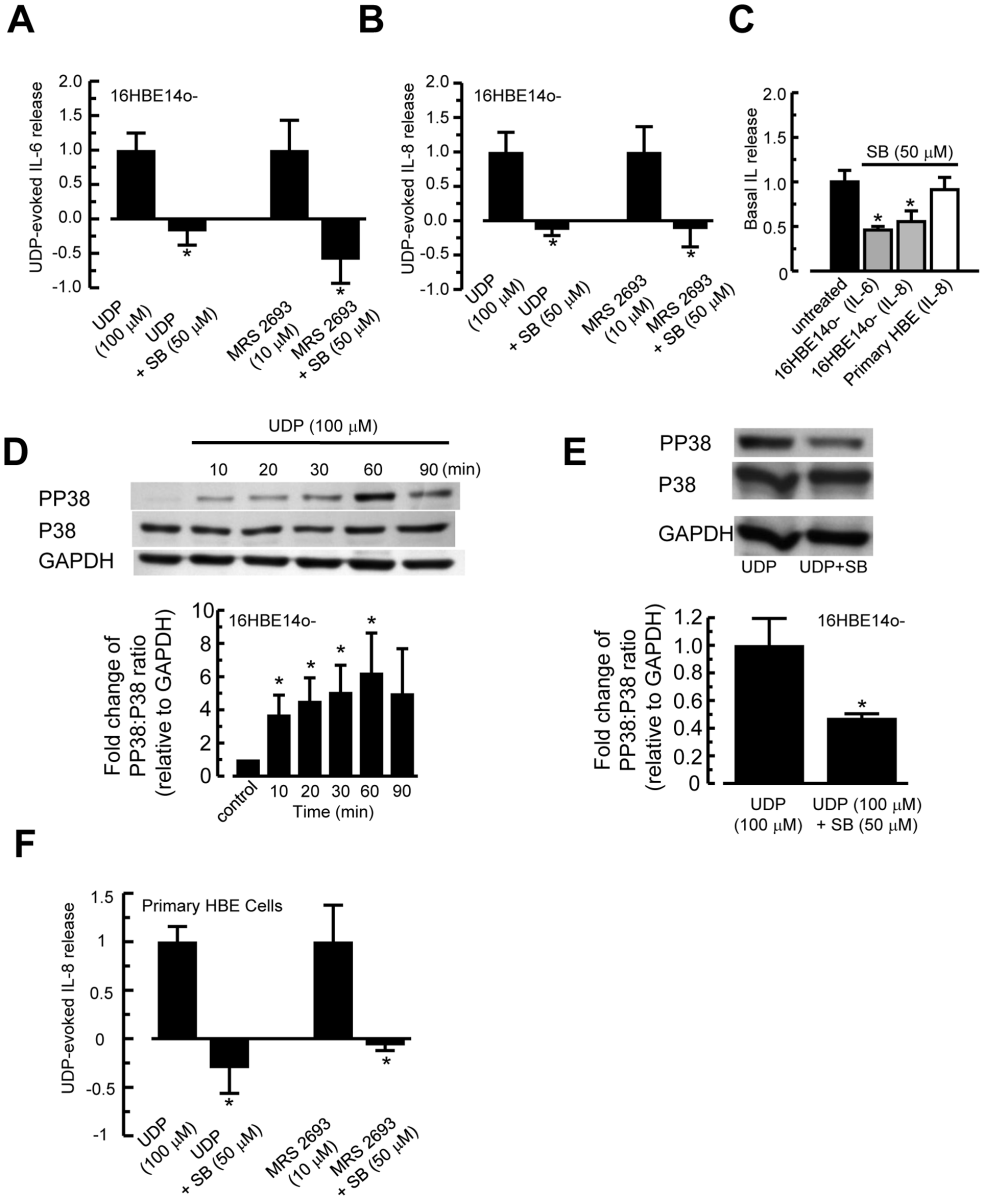
Involvement of p38 MAPK in IL-6 and IL-8 release. (A, B) Cells were treated with UDP alone or co-incubated with p38 MAPK inhibitor, SB 203580 (50 µM), for 6 h. IL-6 (A) and IL-8 (B) levels were measured with an ELISA. Levels of IL-6 and IL-8 were corrected with vehicle control alone and normalized to UDP-stimulated cytokine release. Each column represents the mean ± S.E. (*n* = 4–6). Statistically significant inhibitory effects compared with UDP-treated control are marked with an asterisk (*p<0.05, Student’s *t*-test). (C) Effect of SB 203580 (50 µM) alone on basal IL-6 (16HBE14o- cells) or IL-8 (16HBE14o- and primary HBE cells) release. Each column represents the mean ± S.E. (*n* = 4–6). Statistically significant inhibitory effects compared with untreated control are marked with an asterisk (*p<0.05, Student’s *t*-test). (D) The 16HBE14o- cells were stimulated with 100 µM UDP for the indicated times. Protein matched lysates were analyzed by western blotting with antibodies against phospho-p38 MAPK and p38 MAPK. The graph represents fold change in phosphorylation signal over total MAPK, normalized to GAPDH. The phosphoMAPK∶total MAPK ratio of the vehicle control was arbitrarily set to 1. Data shown are representative of 6–7 independent experiments (*p<0.05 compared with vehicle control, Student’s *t*-test). (E) Similar western blot experiments were performed to show the inhibitory effect of SB 203580 on UDP-stimulated p38 MAPK activation in the absence or presence of the inhibitor (50 µM) for 60 min. Data shown are representative of five independent experiments (*p<0.05 compared with the UDP control, Student’s *t*-test). (F) Similar to (A) and (B), primary HBE cells were stimulated with UDP or MRS 2693 for 6 h in the absence or presence of SB 203580 (*n* = 5–6).

Our previous study demonstrated that p38 MAPK, ERK1/2 MAPK, and NF-*κ*B pathways are involved in poly-L-arginine-stimulated IL-6 and IL-8 release in 16HBE14o- cells. To verify that p38 MAPK is implicated in the P2Y_6_ receptor-mediated IL-6 and IL-8 release, cells were stimulated with UDP (100 µM) or MRS 2693 (10 µM) for 6 h in the presence or absence of a specific p38 MAPK inhibitor, SB 203580 (50 µM). UDP or MRS 2693 stimulated release of IL-6 and IL-8 in the presence or absence of the inhibitor was calculated by subtracting background cytokine concentrations from vehicle-treated control cells, and was expressed as 1. SB 203508 significantly reduced UDP or MRS 2693-induced IL-6 (Fig. 5A) and IL-8 (Fig. 5B) release. A negative ratio indicated that in the presence of the inhibitor, net cytokine secretion was lower than that of control cells, suggesting that SB 203580 alone had an inhibitory effect on constitutive IL-6 or IL-8 release (Fig. 5C). These results indicated that the p38 MAPK pathway is implicated in P2Y_6_ receptor-mediated IL-6 and IL-8 release. To further demonstrate the involvement of the p38 MAPK pathway, 16HBE14o- cells were stimulated for 10–90 min with UDP (100 µM), and p38 MAPK activation was examined by determining the ratio of phosphorylated to total p38 MAPK (Fig. 5D). UDP activated p38 MAPK in a time dependent manner, with a maximum activation after stimulation for 60 min. To further confirm that UDP activated p38 MAPK, cells were stimulated with UDP (100 µM) for 60 min in the presence or absence of SB 203580 (50 µM) (Fig. 5E). SB 203580 significantly reduced UDP induced p38 MAPK activity by 52.6±3.1%. Fig. 5F shows that in primary HBE cells, treating cells with SB 203580 also inhibited the UDP or MRS 2693-induced IL-8 release, suggesting the involvement of the p38 MAPK pathway. Because UDP did not stimulate any significant release of IL-6 in primary HBE cells, we did not perform any studies on the possible involvement of the p38 MAPK pathway in UDP-stimulated IL-6 release.

### Involvement of ERK1/2 in P2Y_6_ Receptor-Mediated IL-6 and IL-8 Release

**Figure 6:**
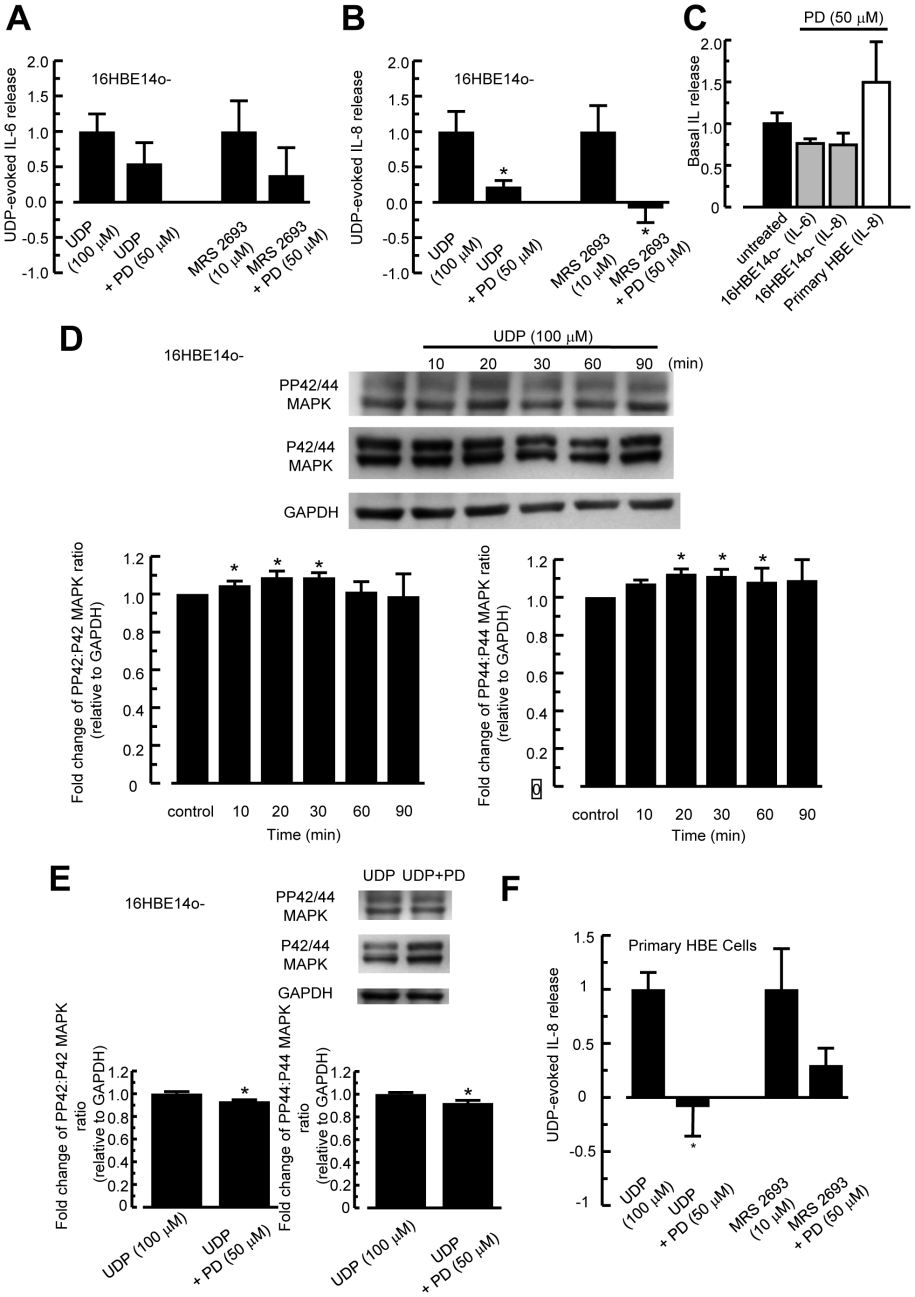
Involvement of ERK1/2 MAPK in IL-6 and IL-8 release. (A, B) Cells were treated with UDP alone or co-incubated with ERK1/2 MAPK inhibitor, PD 98059 (50 µM), for 6 h. IL-6 (A) and IL-8 (B) levels were measured using an ELISA. Levels of IL-6 and IL-8 were corrected with vehicle control alone and normalized to UDP-stimulated cytokine release. Each column represents the mean ± S.E. (*n* = 4–6). Statistically significant inhibitory effects compared with UDP-treated controls are marked with an asterisk (*p<0.05, Student’s *t*-test). (C) Effect of PD 98059 (50 µM) alone on basal IL-6 (16HBE14o- cells) or IL-8 (16HBE14o- cells and primary HBE cells) release. Each column represents the mean ± S.E. (*n* = 4–6). (D) The 16HBE14o- cells were stimulated with 100 µM UDP for the indicated times. Protein matched lysates were analyzed by western blotting with antibodies against phospho-ERK1/2 and ERK1/2 MAPK. The graph represents fold change in phosphorylation signal over total MAPK, normalized to GAPDH. The phosphoMAPK∶total MAPK ratio of the vehicle control was arbitrarily set to 1. Data shown are representative of six independent experiments (*p<0.05 compared with vehicle control, Student’s *t*-test). (E) Similar western blot experiments were performed to show the inhibitory effect of PD 98059 on UDP-stimulated ERK1/2 MAPK activation in the absence or presence of inhibitor (50 µM) for 30 min. Data shown are representative of five independent experiments (*p<0.05 compared with the UDP control, Student’s *t*-test). (F) Similar to (A) and (B), treating primary HBE cells with PD 98059 inhibited UDP, but not MRS 2693-stimulated IL-8 release (*n* = 5–6). The cells were stimulated with the P2Y_6_ agonists for 6 h.

To examine the possible involvement of ERK1/2 in the induction of IL-6 and IL-8 secretion by UDP, similar experiments were performed using PD 98059, a specific ERK 1/2 MAPK inhibitor. Results indicated that 50 µM PD 98059 inhibited IL-8 (Fig. 6B), but not IL-6 (Fig. 6A) induced by UDP (100 µM) or MRS 2693 (10 µM). PD 98059 did not have any basal effect on IL-6 or IL-8 release (Fig. 6C). Western blot data confirmed that UDP could activate ERK1/2 MAPK in 16HBE14o- cells (Fig. 6D), which could be inhibited by PD 98059 (Fig. 6E). There was no detectable change in the p42/44 MAPK ratio. Similarly, ERK1/2 MAPK inhibitor could also reduce the UDP stimulated IL-8 release in primary HBE cells (Fig. 6F). It appeared that PD98059 could also inhibit IL-8 release stimulated by MRS 2693, but this effect was not statistically significant. Taken together, the results suggest that P2Y_6_ receptor-mediated IL-8 release involves the activation of the ERK1/2 MAPK pathway.

### Activation of the NF-*κ*B Pathway by UDP

**Figure 7:**
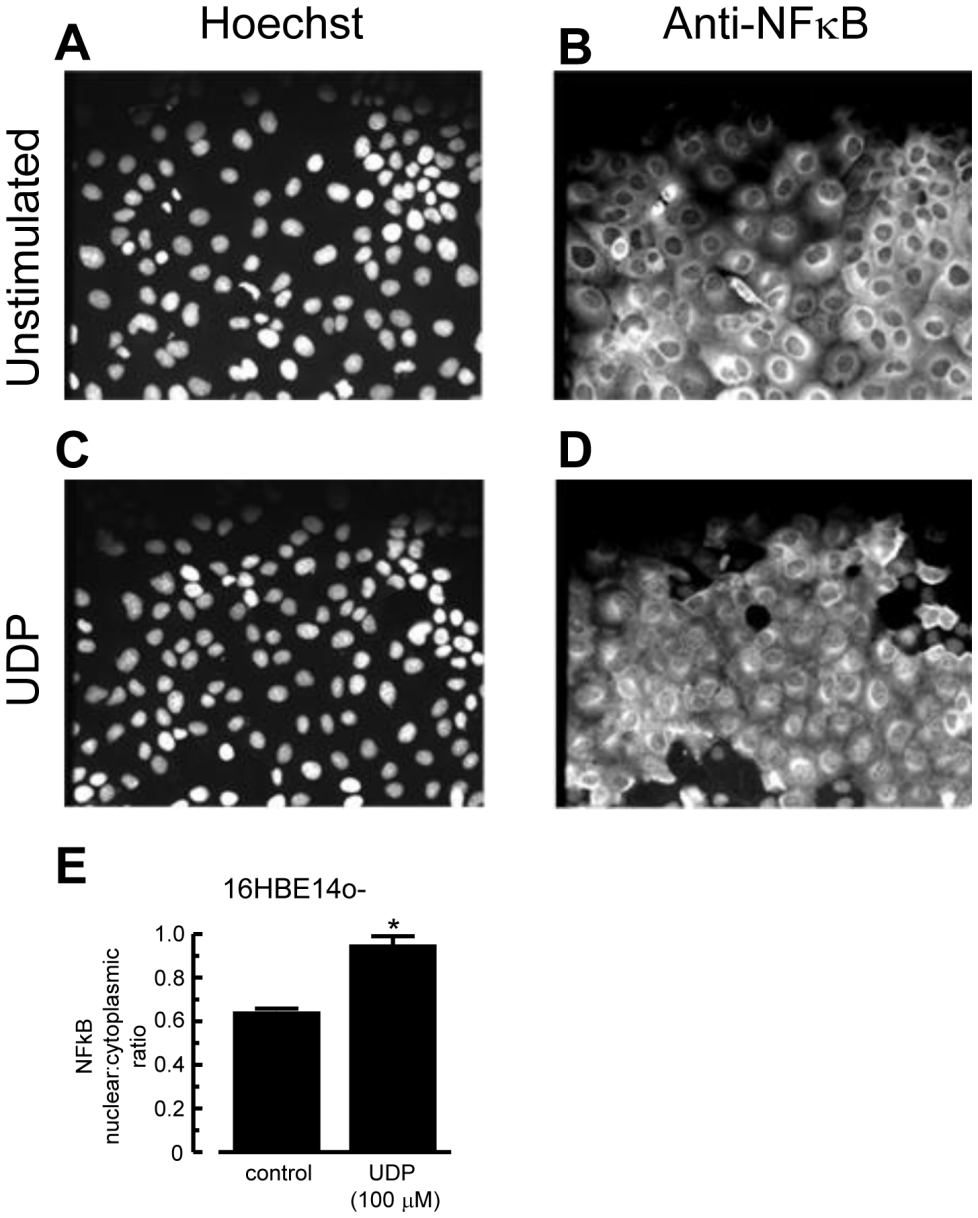
UDP stimulated translocation of NF-κB. The 16HBE14o- cells were stimulated with vehicle control (A, B) and 100 µM UDP (C, D) for 60 min. The nuclei (A, C) were stained with Hoechst 33342, and NF-*κ*B (B, D) was stained with anti-NF-*κ*B and Alexa Fluor 488 goat anti-rabbit secondary antibody (magnification 20×). (E) Immunofluorescence of NF-*κ*B in the nuclear region and cytoplasmic region was quantified using NIH ImageJ software. Nucleus∶cytoplasm ratios of NF-κB staining were calculated. Each column represents the mean ± S.E. (*n* = 3) (*p<0.01 compared with vehicle control, Student’s *t*-test).

To test whether activation of P2Y_6_ receptor could lead to NF-κB translocation, immunofluorescent staining was used. In Fig. 7A-D, UDP stimulated translocation of NF-κB from the cytoplasm into the nucleus. Fig. 7E shows a summary of the image quantification. Upon treating the cells with UDP (100 µM) for 1 h, the nucleus∶cytoplasm ratio increased to 0.95±0.04, while unstimulated cells had a nucleus∶cytoplasm ratio of 0.64±0.01.

### Involvement of Ca^2+^ but not the cAMP/PKA Pathway in IL-6 and IL-8 Release

**Figure 8:**
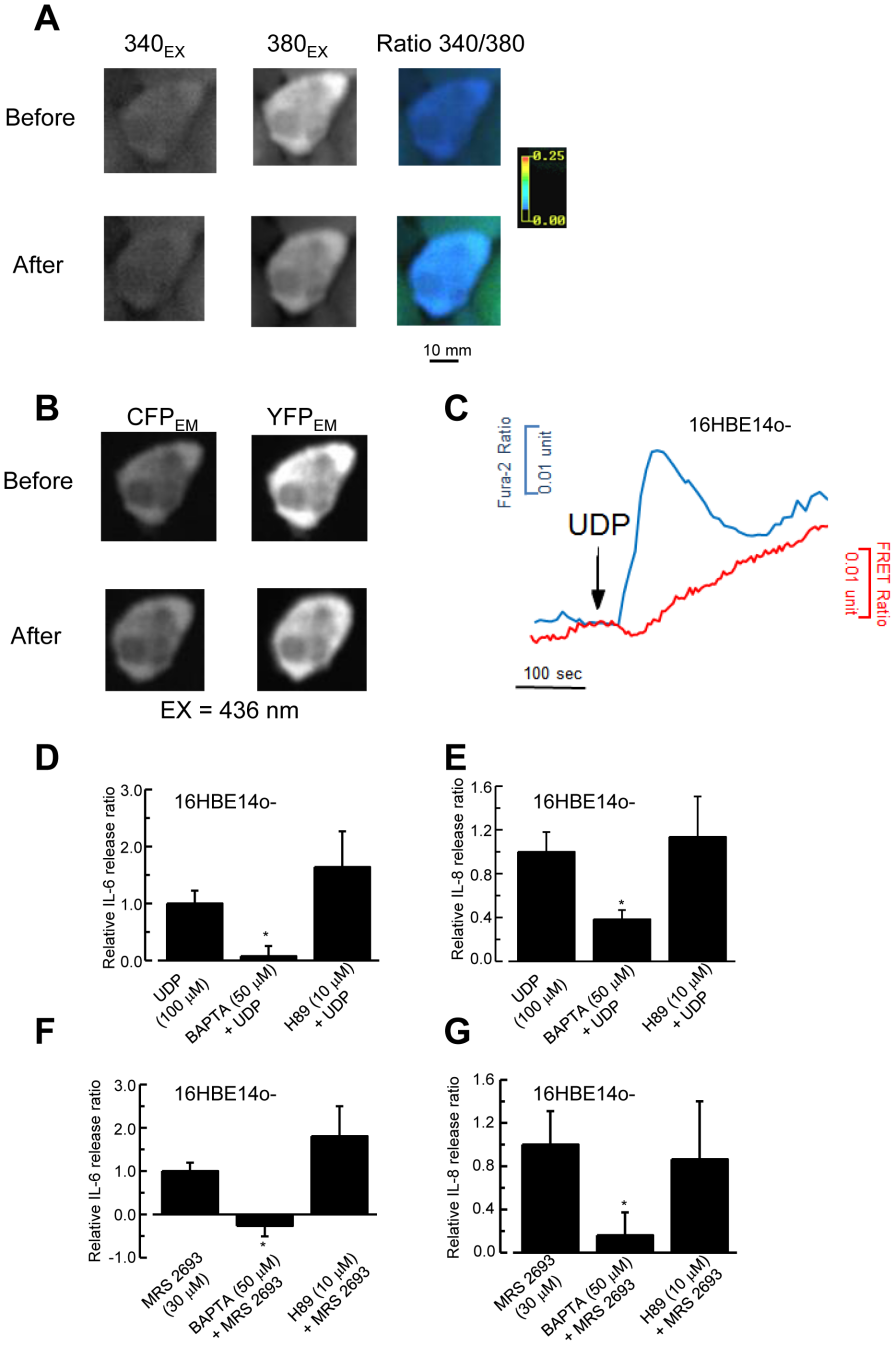
Simultaneous imaging of Ca^2+^ and cAMP signaling, and calcium dependence of UDP-stimulated IL-6 and IL-8 secretion. Digital image of a single 16HBE14o- cell, loaded with Fura-2 (A) transiently expressing the Epac sensor (B) before and after stimulation with UDP. Multiple excitation wavelengths (340 nm, 380 nm, and 436 nm) and emission optical filters were used to resolve Fura-2 and Epac sensor fluorescence as described in Materials and Methods. [Ca^2+^]_i_ was represented by pseudo-colored displays for the Fura-2 ratio (340/380 panel). The monochrome CFP (CFP_EM_) and FRET (YFP_EM_) images show the cytosolic distributions of the fluorescent Epac probe in 16HBE14o- cells transfected with CFP-Epac-YFP. (C) Effect of UDP on cAMP levels (CFP/YFP emission ratio; FRET ratio) and [Ca^2+^]_i_ (Fura-2 ratio) in a single 16HBE14o- cell. The traces are representative of cAMP and Ca^2+^ responses imaged simultaneously in individual cells (*n*≥3 cells for each treatment) in at least three independent experiments. (D, E) The 16HBE14o- cells were co-incubated with UDP (100 µM) in the presence or absence of 50 µM BAPTA-AM or 10 µM H89. Levels of IL-6 (D) and IL-8 (E) were corrected with vehicle control alone and normalized to UDP-stimulated cytokine release. Each column represents the mean ± S.E. (*n* = 5–6). Statistically significant inhibitory effects compared with UDP-treated controls are marked with an asterisk (*p<0.05, Student’s *t*-test). (F, G) Similar experiments were performed to show the inhibitory effects of H89, but not BAPTA-AM on MRS2693-stimulated IL-6 (F) and IL-8 (G) release. Each column represents the mean ± S.E. (*n* = 6–7). Statistically significant inhibitory effects compared with the MRS 2693-treated control are marked with an asterisk (*p<0.05, Student’s *t*-test).

Our previous study demonstrated that stimulation of P2Y_6_ receptor by UDP in 16HBE14o- cells activated both Ca^2+^ and PKA dependent signaling pathways [1]. However, both [Ca^2+^]_i_ and PKA activity were measured in separate experiments, whereas cellular cAMP level was not measured directly. In this study, simultaneous imaging of [Ca^2+^] and cAMP in single polarized 16HBE14o- cells was accomplished by a combination of a dual excitation (Fura-2) and a FRET-based approach using an Epac sensor. Fura-2 (Fig. 8A) and CFP/YFP fluorescence (Fig. 8B) were determined in a single polarized 16HBE14o- cell. Fig. 8C shows the respective tracings of UDP stimulated increase in the Fura-2 and FRET ratios, representing the real-time changes in [Ca^2+^]_i_ and cAMP levels, respectively. Similar results were obtained in three independent experiments. Stimulation of the cells increased FRET and Fura-2 ratios to 0.018±0.005 and 0.028±0.006, respectively. The results further confirmed that stimulation of P2Y_6_ receptor activates both Ca^2+^ and cAMP signaling pathways in 16HBE14o- cells.

To further test whether IL-6 and IL-8 release is dependent on UDP-stimulated Ca^2+^ and cAMP/PKA signaling pathways, we performed experiments using the intracellular Ca^2+^ chelator, BAPTA-AM, and a PKA inhibitor, H89. The 16HBE14o- cells were incubated in BAPTA-AM (50 µM) or H89 (10 µM) and stimulated with UDP (100 µM) for 6 h. As shown in Fig. 8D and 8E, preincubation of epithelia with BAPTA-AM reduced subsequent IL-6 and IL-8 release resulting from UDP stimulation by 92.4±17.8% and 61.6±8.3%, respectively. However, H89 showed no inhibitory effect on UDP-stimulated IL-6 and IL-8 release, suggesting that this effect was mediated via an increase in [Ca^2+^]_i_, but not cAMP. Similar results on the inhibitory effect of BAPTA-AM were obtained for IL-6 (Fig. 8F) and IL-8 (Fig. 8G) release when the cells were stimulated with MRS 2693.

## Discussion

In this study, we have demonstrated a novel pathway for induction of IL-6 and IL-8 expression in human bronchial epithelial cells. UDP released from damaged epithelial cells could induce cytokine secretion through activation of a P2Y_6_ receptor-mediated signaling pathway. Our previous electrophysiological study demonstrated that poly-L-arginine induced a decrease in transepithelial resistance in 16HBE14o- cells. It has been reported that poly-L-arginine would cause membrane damage, resulting in increased permeability, loss of cell-cell contacts and generalized cell damage in 16HBE14o- cells and primary HBE cells. Interestingly, an early study in the 1970’s by Quinton and Philpott had already demonstrated that cationic polymers caused membrane damage to rabbit gall bladder epithelial cells [22]. In this study, we have shown that treatment with poly-L-arginine resulted in cell damage, as judged by increased membrane permeability to LDH. Taken together, it is likely that the UDP release was not due to a regulated mechanism. However, we could not exclude the possibility that UDP may be released via an exocytotic pathway as demonstrated in astrocytoma cells [23].

In our previous study, protein and mRNA expression of P2Y_6_ receptors in 16HBE14o- cells was demonstrated using western blot analysis and qRT-PCR, respectively. The cells also expressed functional P2Y_6_ receptors, which could be activated by UDP to stimulate both Ca^2+^- and cAMP dependent Cl^−^ secretion [1]. In the present study, besides confirming the expression of P2Y_6_ receptors in 16HBE14o- cells, we found that primary HBE cells also expressed a similar pattern of P2Y receptor subtypes, including P2Y_6_. We demonstrated that exogenous application of UDP or MRS 2693 induced the secretion of two proinflammatory cytokines, IL-6 and IL-8, from 16HBE14o- cells. The human P2Y_6_ receptor was identified and characterized as a UDP selective receptor [15], and UDP was a natural ligand of the P2Y**_6_** receptor [24]. MRS 2693 is a synthetic and selective agonist of P2Y_6_ receptor which displays no activity towards other P2Y receptors [25]. Therefore, our data shows that activation of P2Y_6_ receptors expressed in 16HBE14o- cells stimulated IL-6 and IL-8 secretion. Functional P2Y_6_ receptors are expressed in primary HBE cells, which are important in mediating UDP-stimulated Cl^−^ secretion [26]. Our data also demonstrate that the P2Y_6_ receptor agonists, UDP and MRS 2693, stimulated in primary HBE cells a similar fold release in IL-8, but not in IL-6. Differences between primary and cell line cultures in cytokine secretion may suggest that the P2Y_6_ receptor pathway was not coupled to IL-6 release in primary HBE cells. Western blot analysis indicated that both 16HBE14o- and primary HBE cells expressed P2Y_6_ receptor protein at similar levels. However, stimulating the 16HBE14o- cells with UDP (100 µM) caused a significantly larger increase in [Ca^2+^]_i_ than that of primary HBE cells. Because the release of IL-6 and IL-8 are Ca^2+^-dependent, another possibility is that activation of P2Y_6_ receptors in primary HBE cells could not elicit a calcium response sufficient to stimulate detectable IL-6 release, although primary HBE cells were capable of secreting IL-6 when stimulated with poly-L-arginine. Furthermore, treating the cells with BAPTA-AM almost completely abolished IL-6, but not IL-8 release, suggesting that IL-6 release is more dependent upon Ca^2+^ than that of IL-8.

In our studies, UDP was treated with hexokinase and glucose to ensure that it was free from UTP contamination. Although UDP may be degraded to UMP and uracil, to the best of our knowledge, there are no reports demonstrating that these breakdown products of UDP may nonspecifically activate other P2Y receptors and regulate IL-6 and IL-8 production. To confirm the involvement of P2Y**_6_** receptors in poly-L-arginine-induced IL-6 and IL-8 release, a selective P2Y**_6_** receptor antagonist, MRS 2578, was used [27], which showed no activity towards other P2Y receptor subtypes, including P2Y_1_, P2Y_2_, P2Y_4_, and P2Y_11_. MRS 2578 blocked poly-L-arginine-induced IL-6 and IL-8 release in a concentration dependent manner, indicating a role for P2Y**_6_** receptors in mediating IL-6 and IL-8 secretion upon treatment of airway epithelia with poly-L-arginine. There was no additive stimulatory effect of poly-L-arginine and UDP in promoting IL-6 and IL-8 release, further supporting the possibility that poly-L-arginine is mediated via UDP release and the P2Y_6_ receptor pathway.

The surface epithelium is a well-established source of a variety of inflammatory cytokines [28]. Apart from the regulation of ion transport in various epithelia, P2Y receptors have been implicated in various inflammatory disorders [29]. It was shown in a murine model that extracellular ATP triggers and maintains asthmatic airway inflammation by activating dendritic cells [11]. UTP and UDP have been shown to stimulate cytokine release in CD11c^+^ murine dendritic cells [30]. Among other P2Y receptor subtypes, recent evidence suggests a proinflammatory role of P2Y**_6_** in inflammation. Experimental inflammation induced expression of P2Y**_6_** receptors in both mouse and human intestinal epithelia [31]. In active inflammatory bowel disease, P2Y**_6_** expression was highly upregulated in activated CD4^+^ and CD8^+^ T cells infiltrating intestinal mucosa [32]. However, we could not detect any upregulation of P2Y_6_ protein expression when cells were treated with 1 µM poly-L-arginine for 24 h (unpublished data). The presence of functional P2Y**_6_** receptors was a prerequisite for IL-8 production by a monocytic cell line stimulated with bacterial LPS [33] and by human intestinal epithelial cells stimulated with neutrophil derived antimicrobial peptides [31]. P2Y**_6_** knock-out mouse macrophages lost UDP-induced IL-6 production [34]. However, the significance of P2Y**_6_** receptors in airway epithelia during asthmatic inflammation is still largely unknown.

Extracellular nucleotides are released during cell damage, and the subsequent activation of P2X and/or P2Y receptors has been implicated in the pathogenesis of several inflammatory lung disorders, such as asthma and chronic obstructive pulmonary disease (COPD) [35]. Release of nucleotides such as ATP and UTP has been well documented in airway cells [36], astrocytoma cells [23], and glia cells [37]. However, the release of other nucleotides such as UDP has been less well documented, although it has been reported that UDP release and subsequent activation of P2Y_6_ receptor on the endothelium is a key step in vascular inflammation [38]. We have demonstrated previously that in a bronchial epithelial cell model of asthmatic inflammation, 16HBE14o- cells could be “chemically injured” by treatment with poly-L-arginine, a surrogate of the highly toxic cationic protein, MBP [13]. When the cells were exposed to this cationic polypeptide, micromolar levels of released UDP were detected in the extracellular medium of 16HBE14o- and primary HBE cells using a newly developed fluorescent probe, known as Probe-1 [16], which has been used to measure nucleotide release *in vitro* [4]. Although the detailed mechanism of poly-L-arginine-induced cellular damage remains unknown, Shahana *et al*. have reported that poly-L-arginine could induce membrane damage (e.g., formation of pores and increased permeability) in airway epithelial cells [39]. When bronchial epithelial cells were treated with poly-L-arginine, the released UDP could act on P2Y_6_ receptors in an autocrine or paracrine fashion to stimulate IL-6 and IL-8 release from the cells. Our previous study demonstrated polarized secretion of IL-6 and IL-8 into the apical lumen of the epithelial cells that were damaged by poly-L-arginine [13]. The enhanced release of UDP during asthmatic inflammation may trigger both bronchial epithelial proinflammatory cytokines release and facilitate neutrophil migration, because IL-8 can function as a neutrophil chemoattractant and activating factor. Therefore, the released nucleotides may not only act on neighboring epithelial cells to release cytokines, but could also act on other immune cells (e.g., neutrophils and monocytic cells) that express P2Y_6_ receptors and that are recruited to the site of inflammation, forming a cytokine network. However, we cannot exclude the possibility that UTP may also be released and then detected by Probe-1, because the poly-L-arginine-stimulated cytokine release could not be completely abolished by MRS 2578. In addition, UTP may have been degraded to UDP by the action of ectonucleotidase [40]. Although UTP is not a ligand for the P2Y_6_ receptor, it could stimulate cytokine production via other P2Y receptor subtypes, such as P2Y_2_ and P2Y_4_, which are also expressed in 16HBE14o- cells. Moreover, poly-L-arginine could induce a greater amount of IL-6 and IL-8 release when compared to that of UDP. Further experiments are required to delineate the contribution of different nucleotides and their cognate receptors in poly-L-arginine-stimulated cytokine production. Nonetheless, our study provides evidence that P2Y_6_ receptors play an important role in the pathogenesis of asthmatic inflammation.

The activation of P2Y receptors is coupled with the release of IL-6 [41] and IL-8 [42], [43] in bronchial epithelia. The bronchial epithelial cells also respond to other Ca^2+^ mobilizing receptor agonists (e.g., histamine) in the production of inflammatory mediators, such as IL-6 and IL-8 [44], [45]. In this study, we have further confirmed that the P2Y_6_ receptor is coupled to both Ca^2+^ and cAMP signaling pathways by using a simultaneous imaging technique in single cells. Our results demonstrate that IL-6 and IL-8 release is also Ca^2+^-, but not cAMP-dependent, because treating cells with an intracellular chelator, BAPTA-AM, could almost abolish UDP-stimulated cytokine production. In our previous study, P2Y_6_ receptor activation by UDP was coupled to both Ca^2+^- and cAMP-dependent signal transduction pathways, while treating cells with the same concentration of BAPTA-AM (50 µM) could completely abolish the P2Y_6_ receptor-mediated increase in [Ca^2+^]_i_ [1].

Previous reports have shown that the P2Y_6_ receptor signals through ERK1/2 MAPK and NF-*κ*B pathways to increase IL-8 secretion [31], [33]. UDP-induced CCL20 (an antimicrobial chemokine) secretion from human monocytes was dependent on P2Y**_6_** receptors through the NF-*κ*B and p38 MAPK pathways [46]. However, little is known about the regulatory control of P2Y**_6_** receptors on MAPK and NF-κB pathways to produce IL-6 and IL-8 in airway epithelia. Our data demonstrated that UDP-stimulated cytokine release is dependent on p38 and ERK1/2 MAPK pathways. In our study, treatment with p38 MAPK inhibitor, SB 203850, abolished UDP-stimulated IL-6 and IL-8 secretion, whereas ERK1/2 MAPK inhibitor, PD 98059, did not have any significant inhibitory effect on IL-6 release. IL-8 release in primary cultures was also sensitive to PD 98059 treatment. Western blot analysis supported the involvement of p38 in IL-6 and IL-8 release, because UDP could markedly increase p38 MAPK activation lasting for approximately 1 h. However, the same stimulation could only induce a slight, albeit significant activation of ERK1/2 MAPK, which is sufficient to induce the release of IL-8. This is in contrast to poly-L-arginine which could stimulate at least a 2.5 fold increase in ERK1/2 MAPK activation, causing IL-6 release [13]. This discrepancy suggests that the effect of poly-L-arginine on IL-6 secretion could not be solely explained by the release of UDP and activation of ERK1/2 MAPK pathway via the P2Y_6_ receptor, indicating that the role of the P2Y receptor in mediating IL-6 release via ERK1/2 MAPK pathway needs to be further studied.

UDP also stimulated translocation of NF-κB from cytoplasm to nucleus, although its stimulatory effect was not as potent as that of poly-L-arginine [13]. UDP only activated NF-κB in a small percentage of cells, in contrast with a nearly complete and synchronized activation by poly-L-arginine [13]. The relatively weak NF-κB induction by UDP may only result in a moderate transcriptional activation of IL-6 and IL-8. This is similar to that of primary microglia, in which UDP barely activated NF-κB, while P2Y_6_ activation was associated with other transcription factors, such as NFATc1 and NFATc2, to induce chemokine secretion [4]. The involvement of other transcription factors needs further investigation. Besides UDP, other extracellular nucleotides have also been reported to activate NF-κB translocation with varied potencies [47]. Whether p38 MAPK acts upstream of, or parallel to, NF-κB remains unknown. Nonetheless, our results support a key role of UDP and the P2Y_6_ receptor, by coupling to p38 MAPK and NF-κB to stimulate IL-6 and IL-8 release in airway inflammation mimicked by a cationic polypeptide.

In summary, our results support the hypothesis that poly-L-arginine stimulates the release of UDP, which acts on P2Y**_6_** receptors and mediates IL-8 protein release via p38 MAPK, ERK1/2, and NF-κB pathways in human bronchial epithelial cells. IL-6 release is coupled to p38, but not to the ERK1/2 MAPK pathway. Consistent with this possibility, future studies should be directed toward investigating the temporal sequence of signaling events leading to pro-inflammatory cytokine secretion. To the best of our knowledge, this is the first report to show that UDP released from airway epithelial cells could serve as a “danger signal” to induce cytokine production by activation of P2Y_6_ receptors. This novel mechanism of nucleotide release, involving P2Y_6_ receptor activation, may represent an important cellular signaling pathway for regulating asthmatic inflammation in airway cells, suggesting that the P2Y_6_ receptor could be a therapeutic target for developing anti-inflammatory strategies.
